# A Contribution to the Study of Parturient Fever of the Cow1Translated from the Recueil de Médecine Vétérinaire, 1889, for The Journal of Comparative Pathology and Therapeutics.

**Published:** 1890-06

**Authors:** 

**Affiliations:** Utrecht, Holland


### CASES, SELECTIONS, TRANSLATIONS.

### A CONTRIBUTION TO THE STUDY OF PARTURIENT FEVER OF THE COW.[1]

By Professor Thomassen,
Utrecht, Holland.

It is with a certain degree of apprehension that we enter upon the consideration of this question, on which we possess an immense literature in the majority of European languages, and regarding which their reigns, nevertheless, a divergence of opinion such as is unknown in the case of any other malady.

The cause and the nature of parturient fever have up to the present time remained an enigma; we still wait for the *felix qui potuit cognoscere causam.* Notwithstanding this, the diagnosis of the disease is very easy, not only for the veterinarian, but even for

[1] Translated from the Recueil de Médecine Vétérinaire, 1889, for *The Journal of Comparative Pathology and Therapeutics.*

the owner of the animal. The circumstances under which the disease presents itself, the epoch of life at which it is manifested, and the characteristic symptoms that accompany it, make it recognizable at the very outset of its course. Frequently on the arrival of the veterinary surgeon, the farmer, even though he be not very perspicacious, can tell the former that the animal is attacked with that deadly malady which annually claims so many victims among our best milkers.

It is characterized by a state of depression, a paralysis of the nerve centres, particularly of the cerebrum and of certain centres of the medulla oblongata, whence result the loss of the intellectual faculties and sensation, and a paralytic state of certain organs. It is manifested besides by a sudden invasion and a rapid progress, conducting to death or to a prompt recovery without convalescence.

With regard to the causes that can bring about this sudden perturbation in the function of the central nervous system and the lesions which it entails, these as yet remain concealed, as we have just said, from the investigations of even the best authorities who have concerned themselves with the disease.

We make no pretension to wish to resolve this difficult problem, but we simply desire to help, perhaps, to make the task easier for others, by publishing at the outset some views collected in practice which are not included in the ordinary list of what is generally admitted regarding the etiology, symptomatology, progress, and duration of the disease.

Besides some facts emanating from our personal experience, we have at our disposal a score of reports by Dutch veterinary surgeons, addressed to a commission devoted to the study of the disease; and from these we have taken some interesting views, in order to bring them to the knowledge of our foreign colleagues.

In the first place, the denomination parturient fever (*fièvre vitulaire*) seems to us hardly logical, seeing that there never is any question of a febrile state in this disease; and in our opinion that of puerperal apoplexy would provisionally be more rational.

Touching the etiology, there are admitted as *predisposing* causes: That the malady attacks by preference the good milkers, those that have calved from one to four days previously, those that are in high condition, and those in which parturition has been effected without the least difficulty; that ordinarily it shows itself after the third calving, in animals whose placenta has been expelled, and that after a first attack cows retain a certain predisposition for the disease.

The exceptions to these generally accepted rules are not rare, as we shall see.

There have been reported many cases in which the parturition presented some difficulties—at least a dystokia that rendered delivery rather laborious. The disease had been observed by reliable practitioners *before* parturition; on the other hand, it has been seen from the seventh up to the twelfth day after calving. It is generally admitted that the disease is proportionally more grave, and that its progress is the more rapid, the more promptly it appears after calving. The epoch at which the malady arises is, therefore, of the greatest importance from the point of view of prognosis at the outset of the disease.

The question whether the malady can arise *before* parturition is of the highest importance from the pathogenic point of view. We had never had occasion to observe in a pregnant cow symptoms suggesting the existence of puerperal apoplexy, until, in 1886, one of our colleagues was good enough to inform us that he had witnessed parturient fever in a cow in that condition. We eagerly went to see the patient. It was a fat cow eight years of age, almost at term, and which had for some days been at grass. It certainly showed all the symptoms of the disease in question. We were then convinced of the possibility of the fact of which we had previously been in doubt.

We were struck with only one thing, that the temperature remained more elevated than it ordinarily is, viz., 39 to 39.5° C. The cow was slaughtered on the second day, and at the autopsy we found an escape of blood in the shape of a clot in the cavity of the arachnoid, over a great part of the hemispheres, and between the cerebellum and the cerebrum. Could one qualify the case with the name puerperal apoplexy? Different practitioners have assured us that, during a practice of twenty to twenty-five years, they have observed two and three cases of the malady before calving.

The animals are most predisposed to contract the disease after having given birth to the 4th–7th calf—the epoch at which the lacteal secretion has generally attained its maximum.

In the reports mentioned above we find related a single case of a primipara attacked with the disease. The cases observed in cows of a very advanced age, even 12 to 15 years, are more frequent.

Frequently parturition was not completed at the moment when the disease made its appearance, and in a few cases deliverance was not practised until after the recovery of the patient.

In some animals attacked after several consecutive births an individual predisposition incontestably exists. Thus we have observed the disease in three successive years in a cow which finally succumbed to it. A colleague reports the interesting fact of an animal that was thrice re-attacked, and which afterward had four calves.

The generally admitted heredity we regard rather as a predisposition of race. That is the case notably with those that furnish the best milkers. In support of this fact we may cite the following example: In the district in which we have practised, milk fever was absolutely unknown forty years ago. Practitioners—veterinary surgeons as well as empirics—have often assured us that at a particular date they found themselves engaged with a malady that had hitherto been completely unknown to them. This epoch corresponded with that at which, in order to improve the race of the country, they had begun to import from the northern provinces animals belonging to the Dutch race properly so-called, renowned as *par excellence* the breed for milking.

It is probable that in other countries—notably in Italy, Spain, America, and even in France—the disease is also more frequently observed since the Dutch race has been imported there. We believe even that in certain districts of these southern countries the disease was until a recent period unknown.

Sudden transference to better feeding, especially if the change takes place shortly before parturition, seems to predispose the animals. Regarding changes in the hygienic conditions, experience has taught us that cows which, having already been for some time at grass, are brought into the house again some days before parturition, often contract the disease when the age and other circumstances predispose them to it.

*Symptoms.*—We have no intention of giving a complete description of the symptoms, which are sufficiently well-known and described in many places. Our intention is simply to refer to certain details of symptomatology.

The disease generally begins with a state of rather pronounced depression. Exceptionally, the patients show over-excitement, which may even amount to furious delirium. According to certain practitioners, the animals sometimes lowed, struck with their horns, their eyes expressed mental disturbance, and often they exhibited even convulsive movements.

But these symptoms soon give way to the ordinary somnolence and a more or less general paralysis.

In the recumbent animal—it may be in the sternal position, able at most to drag itself along the ground, or, in the graver cases, extended full length on the litter—the temperature, pulse, and respiration merit in the first place special attention.

The rectal temperature is generally under the normal when the disease has reached the comatose period. It may happen that the thermometer has mounted at the outset to 39-39.5° C., descending after some hours to 38° and even 36° C., remounting afterward when an amelioration is produced in the animal's condition. In the report cited we find notes of 32° and 32.2°. In the last case observed by a distinguished practitioner, the temperature remounted after three hours to 37.8°, and the animal recovered. In our opinion, it is necessary to mistrust excessively low figures, which we regard as the consequence of a paralysis of the sphincter of the anus, giving access of air to the rectum, and thus lowering the temperature in that part. Another practitioner claims to have seen an elevation of temperature from 39° to 43° in consequence of exaggerated application of woolen coverings.

The pulse is always more frequent than in the normal state. As a mean, one may count 60 to 80 beats; even 100 to 120 pulsations have been noted. At the beginning of the disease it may be very large and soft, and in proportion as the condition becomes aggravated it becomes smaller, often even imperceptible, and sometimes irregular.

Respiration is generally slow and deep, unless complications on the part of the lungs are already present. In grave cases the respiration may be only eight to five per minute.

Among the paralytic symptoms those resulting from a paralysis of the pneumogastric in the first place merit our attention. It is denoted by a paralysis of the pharynx, œsophagus, rumen, and reticulum, and by the loss of sensibility in the mucous membrane of the larynx, and a paralysis of that organ. A greater frequency of the cardiac contractions and a slowing of the respiration are equally the consequence of it. These same symptoms are observed after section of the pneumogastrics in ruminants. Besides the difficulty in deglutition (dysphagia), the paralysis of the œsophagus is manifested by a sound during swallowing—that is to say, a gurgling sound (*bruit de glou-glou*)—which is audible along the whole length of the neck during the administration of liquids. In consequence of the loss of contractile power of this organ, liquids pass it as by an inert tube.

From the pharyngeal and laryngeal paralysis there often results a penetration of foreign bodies (food or medicines) into the air passages, giving rise to a pneumonia. We may note that the alimentary matters encountered in the respiratory organs often come from the rumen, whence they have been rejected into the pharynx, afterward entering the trachea.

A snoring sound often heard in grave cases results from paralysis of the soft palate. Other organs, such as the inferior maxilla, the tongue, the upper eyelids, are often more or less paralyzed. The cornea has generally lost its sensibility, and its surface, which has lost its polish, seems turbid. The eyeballs are generally turned downward. These symptoms may in great part be considered as the result of paralysis of the trigeminal and hypoglossal nerves. The influence of nervous troubles on the digestive organs is manifested by indigestion—an obstinate constipation, with complete absence of evacuations, and frequently with tympanites. According to the view of some practitioners, this last complication ought to be regarded as dangerous when it appears at the outset of the disease. At a more advanced stage, and especially in Winter, it offers less danger. The distention often disappears on changing the position of the animal, by laying it flat on its right side. Micturition hardly ever takes place spontaneously. Since M. Nocard fixed attention on the symptom, the presence of sugar in the urine has generally been observed, and also a certain amount of albumen. The lacteal secretion, although considerably diminished, is not generally interrupted, and the udder retains almost its normal volume. The duration of the disease may vary from one to three days. One case was reported (*loc. cit.*) where the malady persisted for seven days. The cow made a half recovery on the third day, had a relapse, but finally recovered completely. Recovery generally takes place without convalescence. In other cases certain complications—among others, monoplegiæ—persist for some time after the general disturbance has disappeared. We have had occasion to observe paralysis of a fore limb, with muscular atrophy, which lasted for about six weeks. Paralysis of a hind limb and paraplegia have been signalized by several practitioners. Frequently these are incurable, and the proprietor resolves on slaughter. Paralysis of the œsophagus, lasting for some days, is also seen. A Dutch veterinary surgeon reports a case of a cow with dysphagia which died in consequence of the arrest of a mouthful of hay in the œsophagus. A complication of puerperal apoplexy mentioned nowhere is gangrene of some of the extremities, this being often observed in some parts of Holland.[1]

In the animal from ten to fifteen days recovered from the disease one suddenly sees fetid matter exuding between the claws and around the coronets of the hind feet. Soon a line of demarcation forms at the middle of the metatarsus, and the entire extremity appears completely sphacelated. Ordinarily the owner decides upon slaughter at the first symptoms, without waiting for the fatal issue.

Dry gangrene (mummification) of the teats is also seen from time to time.

*Nature of the Disease.*—What is the cause and what are the lesions which can engender the sudden and grave functional disturbances of the nervous system which we have just enumerated?

Regarding the predisposing causes, there is, as we have seen, general agreement, but concerning the direct or efficient cause there is great divergence of opinion, and many hypotheses have been emitted on the subject. We proceed to cite summarily the principal of these.

The published theories regarding the nature of milk fever may be divided into two groups :

1. Those that admit circulatory disturbance in the nervous centres, either anæmia or hyperæmia (inflammation).

2. Those that suppose the presence of foreign substances in the blood current, exercising a pernicious influence on the central nervous system.

I. Anæmia. Haubner admitted as the cause of the disease a

[1] About two years ago a case of this nature was brought under our notice by Mr. J. R. U. Dewar, Aberdeen.—Ed.

cerebral anæmia resulting from a hyperæmia *ex vacuo* of the abdominal organs. He attributed the predisposition to this congestion in cows of a certain age to the loss of the contractile power of the abdominal walls which exists in young animals. Werner (1868) and Prehr likewise maintained that the cerebral anæmia arose in consequence of an abdominal congestion.

Billings (*American Journal of Comparative Medicine*, 1884) supposed that an exaggerated excitability of the nervous apparatus of the uterus provoked in a reflex way a spasm of the small arteries of the cerebrum and kidneys, and, as a result, anæmia.

Franck believed in an acute secondary anæmia, succeeding a cerebral congestion, which develops in the following manner :— In animals that have easily given birth to their calves the uterus contracts, and its capacity is suddenly diminished by the *post-partum* pains, occasioning a grave perturbation of the circulation. The contracted uterus will receive less blood, and in ordinary cases this is counterbalanced by an afflux to the mammary glands and skin. A lowering of the temperature of the skin may hinder this physiological compensation, the blood will go elsewhere, and a cerebral congestion will be a consequence of this perturbation. From the hyperæmia there very soon results a cerebral œdema, and hence, by compression of the vessels, an anæmia. A predisposition to serous exudation exists in consequence of the hydræmic state of the blood in animals after parturition, especially when, during the latter period of gestation, they have been attacked with a passive congestion of the kidneys and albuminuria (?).

Franck regards the very great frequency of cerebral hyperæmia in the cow as a consequence of the division of the internal carotid into a certain number of small vessels before its entrance into the cranium, and the formation in the cerebral cavity of an intricate network (*reseau admirable*), from which arises a common trunk to furnish the cerebral arteries. Franck's theory is defended in the second edition of his treatise on obstetrics by Göring, of Munich (1887).

Walley (1874) had already sought relations between milk fever and the special division of the arteries in the *bovidæ*. This *rete mirabile* exists in the pig also, and a disease which is frequently seen in the north of Germany in that species after parturition is considered by certain authors as identical with that of the cow.[1]

[1] Notable differences are that in the pig the paralysis is not complete, that there is always intense fever, and that the patients generally recover.

II. HYPERÆMIA. Festal, in a memoir addressed to the Société Centrale maintained that in several cases he had found blood clots under the arachnoid. He regarded plethora as the cause.

Noquet (1853) also admitted a congestion of the cerebro-spinal system and splachnic nerves, resulting from blood plethora, repletion of the stomach at the moment of parturition, and intensity of the fever of lactation.

According to M. Sanson (*Journal des Vétérinaires du Midi*, 1854), the disease is the consequence of a sudden disturbance in the physiological state of the womb after parturition, consisting in the abrupt departure of blood. The sanguineous fluid distributed in the organs of gestation is suddenly expelled, and it distributes itself in the other organs, and especially in the nervous centres, this being favored by cooling of the skin. This theory has a great analogy with that of Franck, which is generally adopted in Germany, and to which it is posterior.

Ayrault (1857) was of the opinion that a cerebro-spinal congestion is determined by lowering of temperature acting directly on the womb, from which the blood is expelled.

Félizet (1866) attributes the congestion of the nervous centres to a moral affection resulting from the immediate withdrawal of the calf from the mother's sight after parturition.

According to M. Violet, an easy parturition would suddenly lower the intra-abdominal pressure, which, during gestation, makes itself felt as far as the heart. During gestation, says M. Violet, the heart was accustomed to bear the compression which for it, as for all the organs contained in the thorax and abdomen, results from the progressive development of the uterus; it had insensibly accommodated itself to this state, and the vessels acted in unison with it. Parturition being effected in a manner instantaneously, the heart suddenly acts with a freedom to which the capillaries are not accustomed. In a measure surprised, the latter allow themselves to be gradually distended, and congestion is produced. Vascular ruptures and consequent hæmorrhages are the possible results. The hæmorrhage accounts for the fatal termination and the rapidity with which the disease sets in.

Gerrard (*The Veterinarian*, 1879) also says that in fifteen cases out of twenty, he succeeded in demonstrating the existence of a sanguineous apoplexy (hæmorrhage). Simonds (*The Veterinarian*, 1880) defended this theory as early as 1840.

According to M. Trasbot, the affection is provoked by congestion of the medulla, with consecutive paralysis.

Among the numerous theories admitting the absorption or direct introduction of foreign products into the blood stream we will cite:

1. Metastasis, that is to say, the absorption of milk into the blood, hence the name "milk fever" employed in some countries. Peasants and cow-dealers generally thus explain the origin of the disease.

2. Absorption of septic products formed in the uterus, from *lochia*, blood clots, or tissue *débris*, exercising a depressing action on the central nervous system. This hypothesis has been supported by Stockfleth, Lanzilloti-Buonsanti, Zundel, Raynaud, etc.

3. Lafosse, in his treatise on pathology, explains the development of the disease in the following manner. The disease results from the white milky juice secreted by the cotyledons, and absorbed by the chorionic villi, not being any longer separated from the blood by the action of the placental glands after parturition; the elements of this fluid thus remain in the blood, and accumulate there until the moment when the mammary glands determine their consumption. When these glands rapidly effect this separation, the febrile movement is unperceived or almost nothing; but if the secretory function of the mammary glands operates slowly, then there arises a more or less intense morbid disturbance, depending, without doubt, on the presence in the blood of a product foreign to its normal composition. The rapid disappearance of the disease as soon as milk is secreted in abundance supports this theory, which, nevertheless, requires to be controlled by analysis of the blood.

4. Hartenstein attributes the disease to absorption of certai products formed in the muscular layer of the uterus, and especially to uric acid formed during the act of parturition.

5. Abadie (1874) admitted at the outset an indigestion with overfilling of the stomachs with food, accompanied by the production of a great quantity of mephitic gases by fermentation, such as carbonic oxide, and carburetted and sulphuretted hydrogen. In short, milk fever would be the result of a mephitic poisoning provoked by the absorption and penetration into the blood current of gases that entail disturbances of the functions of all the organs.

6. Carsten-Harms, of Hanover, considered the disease an anæmia, resulting from the introduction of air by aspiration through the uterine vessels after detachment of the placenta. He maintained that he was able to observe the presence of air in the cerebral vessels.

7. Schmidt-Mülheim, in 1885, published a theory which has also been adopted by Friedberger and Fröhner in their treatise on pathology.

He compares the disease to the *botulisme* observed in man after the ingestion of sausages arrived at a certain degree of decomposition, giving rise to ptomaïnes. These alkaloids of animal origin provoke a paralysis of the muscles of the tongue, pharynx, palate, œsophagus, larynx, eyes, etc., and even of the intestinal organs and vessels. The author believes that he has seen the same series of symptoms in parturient fever as in the precited intoxication in man.

The toxic agent is supposed to be formed in the albuminoid products contained in the closed uterus under the form of *lochia*.

Certain authors, such as Rychner, Binz, Köhne, Röll, Fuchs, etc., admit as the starting point a paralysis of the ganglionic system, afterward extending to the medulla and cerebrum.

We pass in silence over the other theories, such as those admitting a leucocythæmia, a superabundance of nervous influence, etc.

To criticise these different theories would carry us too far. In our opinion none of the hypotheses mentioned is based on facts sufficiently established at the autopsy, or acquired by way of experimentation. We have only the cerebral œdema, encountered constantly according to certain authors, and the hæmorrhage of that organ, according to others, invoked as proof in support of these theories.

Remaining always in the realm of hypotheses, we submit, as conditions which any theory explaining the genesis of puerperal apoplexy must satisfy, the following:

It must demonstrate:—1. Why the disease attacks exclusively the *bovidæ*, and none of the other domestic females, finding a condition existing in this species and absent in the others.

2\. Why it is observed only in animals of good milking breeds, well-nourished, of a certain age, and in which parturition has presented little or no difficulty.

2\. Why the predisposition is greatest from the second to the third day after parturition, since it is then that the disease generally shows itself; and why it may exist, although rarely, even before parturition.

4\. It ought to explain the sudden invasion of grave symptoms, and their disappearance as suddenly without a period of convalescence.

With regard to the latter fact, it is necessaiy to exclude, in our opinion, anatomical lesions of the nervous centres, the disappearance of which so suddenly is hardly admissible. In the cases where they have been discovered at the autopsy, they must be considered as purely secondary. For a number of years we have regarded the functional disturbance of the central nervous system in this disease as resulting from the action of a chemical agent entailing often a rapid death, or, as in any other intoxication, even of the most active agents, ending in a recovery characterized by a sudden transition from a state of complete paralysis of the senses to a state of perfect excitability.

What is the toxic agent, where can it develop in the cow, and why specially in the conditions which we have enumerated? These are so many questions on which we propose to return.

